# FLT3-inhibitor therapy for prevention and treatment of relapse after allogeneic hematopoietic cell transplantation

**DOI:** 10.1007/s12185-022-03352-6

**Published:** 2022-04-23

**Authors:** Francesca Biavasco, Robert Zeiser

**Affiliations:** Department of Internal Medicine I, Hematology, Oncology and Stem Cell Transplantation, University Medical Center Freiburg, University Hospital Freiburg, 79106 Freiburg, Germany

**Keywords:** Relapse, Acute myeloid leukemia, Allogeneic hematopoietic stem cell transplantation, FLT3-ITD, Midostaurin, Sorafebin, Gilteritinib

## Abstract

The curative potential of allogeneic hematopoietic cell transplantation (allo-HCT) for acute myeloid leukemia (AML) relies on the graft-versus-leukemia (GVL)-effect. Relapse after allo-HCT occurs in a considerable proportion of patients, and has a dismal prognosis with very limited curative potential, especially for patients with FLT-ITD-mutated AML. Since the first description of sorafenib for treatment of FLT3-ITD-mutated AML, several clinical trials have tried to determine the efficacy of FLT3 inhibitors for preventing and treating AML relapse after allo-HSCT, but many questions regarding differences among compounds and mechanisms of action remain unanswered. This review provides an overview on the established and evolving use of FLT3 inhibitors to prevent or treat relapse of AML in the context of allo-HCT, focusing on the recently discovered immunogenic potential of some FLT3 inhibitors and addressing the possible mechanisms of leukemia drug-escape.

## Introduction

Acute myeloid leukemia (AML) harboring an activating mutation in FLT3 represents around one third of AML cases and is characterized by high relapse rate and dismal prognosis despite undergoing allogeneic hematopoietic cell transplantation (allo-HCT) [1, 2].

**Fig. 1:**
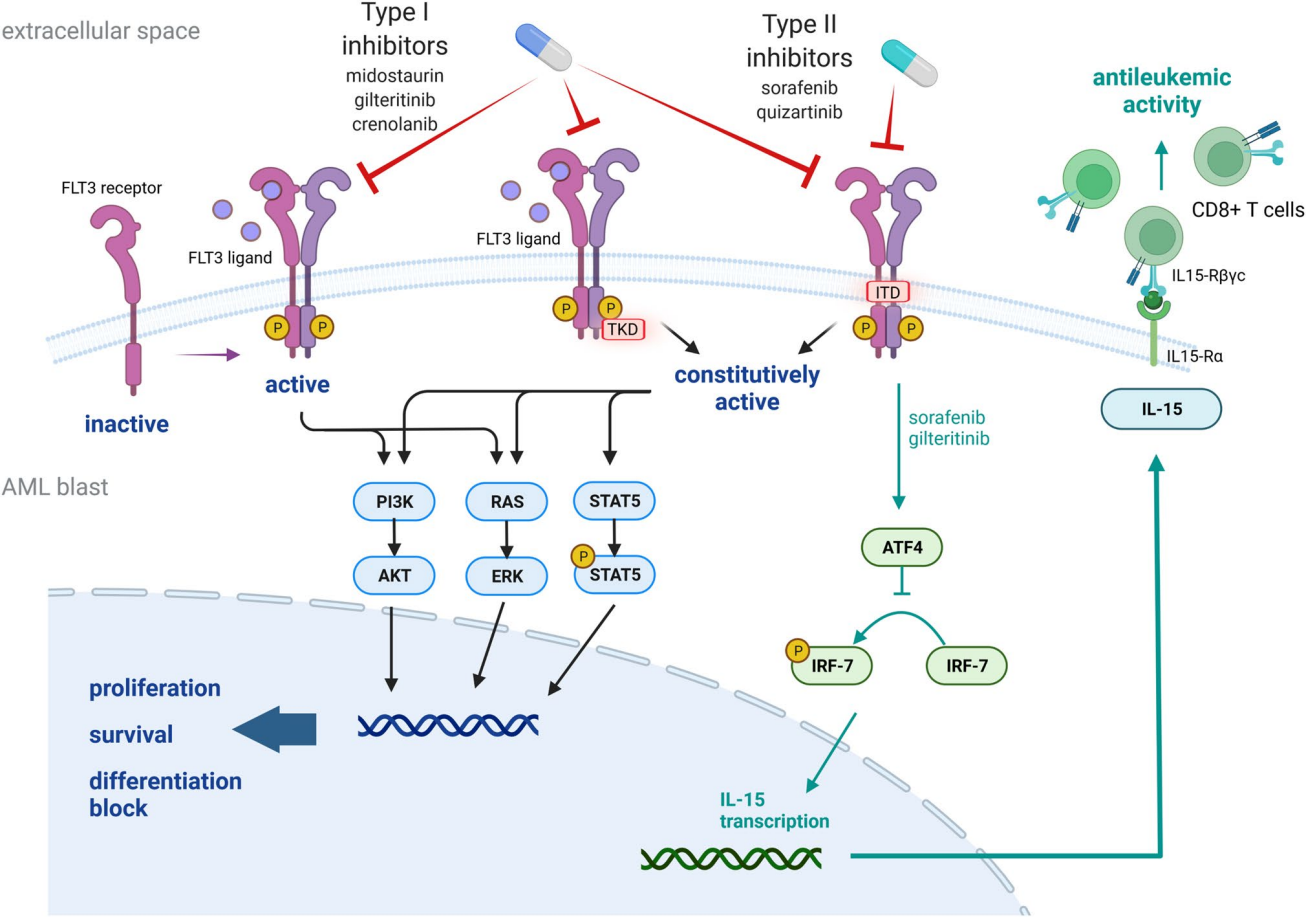
Mechanism of action of type I and type II FLT3 inhibitors and downstream effects. Proposed mechanism through which sorafenib/gilteritinib leads to increased IL-15 transcription. Inhibition of FLT3 receptor tyrosine kinase signaling reduces ATF4 production. Reduced ATF4 levels result in less inhibition of IRF7 phosphorylation and activation. Active p-IRF7 can translocate to the nucleus, where it activates IL-15 transcription. IL-15 activates CD8 T cells and NK cells.

FLT3 is a transmembrane receptor tyrosine kinase physiologically expressed by hematopoietic stem and progenitor cells. The most common mutation is an internal tandem duplication (ITD) within the juxtamembrane domain, which constitutively activates the receptor, promoting proliferation and cell survival mainly through the activation of the PI3K, MAPK (ERK) and STAT5 signaling pathways (Fig. 1). The second type of mutation is a single amino acidic exchange in the tyrosine kinase domain (TKD), resulting in the loss of auto-inhibitory function [3–7].

Mutations within FLT3 occur late in leukemogenesis, are strong driver mutations and have been identified as a druggable target in FLT3-mutated AML [8, 9]. Efforts to establish FLT3 tyrosine kinase inhibitors (TKI) as treatment options are now spanning over two decades and did not yield lasting clinical benefits as monotherapy at first [10–12]. It was only recently that the multi-kinase inhibitor midostaurin in combination with chemotherapy and giltieritinib as monotherapy demonstrated clinical benefit in newly diagnosed FLT3-mutated AML and in relapsed/refractory disease respectively, leading to their FDA and EMA approval [13, 14].

FLT3 inhibitors are classically divided in first-generation multi-target inhibitors (such as midostaruin, sunitinib and sorafenib) and next-generation selective inhibitors (such as quizartinib, crenolanib and gilteritinib). Furthermore, inhibitors can be classified based on the ability to bind to the active conformation of the receptor (type I inhibitors, such as midostaurin, crenolanib, gilteritinib, sunitinib), which are functional against FLT3-ITD and TKD-mutated forms or to bind only the inactive or ITD-mutated conformation of the target (type II inhibitors, such as sorafenib, quizartinib), which are therefore intrinsically inert toward TKD-mutated receptors [15]. Since one major mechanism of resistance to type II inhibitors is the acquisition of additional FLT3 TKD mutations, the use of type I inhibitors is currently favored by clinicians [16–19]. Interestingly, some durable remissions were observed in patients with FLT3-ITD-mutated AML treated with sorafenib after allo-HCT. Further analysis on this synergism leads to the discovery of the immuno-stimulatory effect of several FLT3 tyrosine kinase inhibitors (FLT3-TKIs), such as sorafenib, tandutinib, midostaurin, crenolanib, quizartinib, through induction of IL15-production in the AML cells (Fig. 1) [20]. The connection between FLT3 inhibition and IL-15 production in the GVL context was reproduced by the group of Teshima using gilteritinib [21].

This review provides an overview on the established and evolving potential use of FLT3 inhibitors to prevent or treat AML relapse in post-transplantation context and addresses pending questions regarding its immunogenicity and the mechanisms of leukemia relapse during or after treatment.

## Treatment of overt relapse

Relapse incidence is highest within the first 2 years after allo-HCT and survival is better if incipient relapse is detected earlier. Therefore, regular monitoring for MRD markers including chimerism is recommended [22, 23]. In overt AML relapse, the reduction of immunosuppression can result in response and even complete remission. Several other strategies exist to “actively” treat AML relapse after allo-HCT. Data on comparison of different strategies for treatment of overt relapse are sparse, but in the last decade, several retrospective studies and clinical trials compared treatment with FLT3-TKI monotherapy with standard chemotherapy, unfortunately none of them focusing on relapse after allo-HCT. A list of the phase II and III clinical trials on FLT3-TKI monotherapy in the context of relapse/refractory leukemia can be found in Table 1.

**Table 1:** Phase II and III trials on FLT3 inhibitor monotherapy in relapse/refractory AML

Inhibitor	Phase	Reference	N° of pat	N° of post HCT	Treatment	Response	Median OS
Quizartinib	Phase II	Cortes 2018 NCT01565668	76	21	Quiz. 30 or 60 mg	ORR 65.8% CRc 47.4%	22.6 weeks
	Phase II	Cortes 2018 NCT00989261	333 A: ≥ 18 years B: ≥ 60 years	88	Quiz. ♂135 mg, ♀90 mg	A: ORR 77% CRc 56% B: ORR 74% CRc 46%	A: 25.4 weeks B: 24 weeks
	Phase II	Takahashi 2019 NCT02984995	37	5	Quiz. 20, 30 or 60 mg	ORR 77.8% CRc 53.8%	34.1 weeks
	Phase III (QuANTUM-R)	Cortes 2019 NCT02039726	367	89	Quiz. 60 mg vs salvage CT	CRc 48% vs 27%	6.2 vs 4.7 months (*p* = 0.02)
Gilteritinib	Phase I–II	Perl 2017 NCT02014558	252	73	Gilt. 20–450 mg	ORR 40% CRc 30%	25 weeks
	Phase III (ADMIRAL)	Perl 2019 NCT02421939	371	26	Gilt. 120 mg vs salvage CT	CRc 54.3% vs 21.8%	9.3 vs 5.6 months (*p* < 0.001)

*pat.* patients, *Quiz.* quizartinib, *Gilt.* gilteritinib, *ORR* overall response rate, *CRc* composite complete response

### Sorafenib

Sorafenib, a first-generation multi-target kinase inhibitor approved for treatment of advanced renal and hepatic cell carcinoma, has been studied since more than a decade in FLT3-ITD-mutated AML patients. First results of small retrospective studies and case series of sorafenib monotherapy in relapse/refractory AML were controversial [24–26]. Metzelder and colleagues reported a composite complete response (CRc) rate of 38% in 65 patients with FLT3-ITD AML refractory to multiple therapy lines, with even more striking effects in patients relapsing after allo-HCT compared to relapse after chemotherapy only [27]. In a retrospective cohort of 29 FLT3-ITD-positive AML patients, who relapsed after allo-HCT, 6 patients (21%) achieved sustained CR with sorafenib monotherapy [28]. Excluding one patient who received a second allo-HCT, four of these patients are in treatment-free remission for a median of 4.4 years. With a median follow-up after relapse of 7.5 years, these data suggest for the first time that FLT3-ITD inhibition alone can induce long-term disease control and conditional cure in patients relapsing after allo-HCT [28]. These results were confirmed by further case series and retrospective studies in patients with FLT3-ITD AML relapse post allo-HCT, who showed impressive responses to sorafenib in individual patients including long-term survival [27, 29–31].

As donor lymphocyte infusions (DLI) and hypomethylating agents are widely used for the treatment of post-transplant AML relapse, some patients received sorafenib in combination with DLI or azacytidine [32]. Treatment of FLT3-ITD-mutated AML relapse post-allo-HCT with sorafenib and DLI was superior to DLI alone and to combination of chemotherapy and DLI in a retrospective analysis [20]. More recently, the triple combination of sorafenib chemotherapy and DLI was shown to be superior in terms of response rate and overall survival compared to the combination of sorafenib and chemotherapy or chemotherapy and DLI (CR 70.7% with triple combination, 50.0% with sorafenib + chemotherapy and 35.3% chemotherapy + DLI, *p* = 0.007) (1 year OS 53.2% with triple combination, 25.0% with sorafenib + chemotherapy and 23.5% chemotherapy + DLI, *p* = 0.003) [33]. Interestingly, the combination of sorafenib and DLI did not show a significant increase in incidence of acute and chronic GvHD [33]. Despite the promising data derived from retrospective analysis on the use of sorafenib combined with DLI for hematological relapse of FLT3-ITD AML post-allo-HCT, there is no prospective trial that would justify this approach in all patients and prevention of relapse rather than treatment would be desirable.

### Quizartinib

As first on the market next-generation potent FLT3-inhibitor [34], quizartinib was intensively studied in relapse/refractory AML. After determination of feasibility and maximal tolerated dose in two phase I trial, including a total of 16 patients after allo-HCT [35, 36], three phase II trial with different dosages were conducted (Table 1). In all phase II trial, a consistent composite complete response in 47–56% of patients was observed and a median overall survival (OS) of 21–34 weeks was reached. These clinically relevant results were confirmed by a large multicentre randomized controlled phase III clinical trial (QuANTUM-R) on 367 patients. Relapse/refractory patients treated with quizartinib monotherapy showed a higher CRc rate (48% vs 27%) and a higher OS (median 6.2 months vs 4.7 months) compared to salvage chemotherapy group [37]. Unfortunately, even though 61 and 28 patients relapsing after allo-HCT were included in quizartinib and chemotherapy group respectively, no subgroup analysis was conducted and a previous allo-HCT did not show a significant benefit for the treatment with quizartinib. Another main downside of this study is the exclusion of patients previously exposed to FLT3-TKI. Since the introduction of midostaurin as standard of care induction therapy in FLT3-ITD mutant AML, such a TKI-free FLT3-ITD-mutated AML population is not representing the real-world population. Quizartinib was approved in Japan in 2019 for the treatment of relapsed/refractory AML based upon promising initial results. In the same period, though, the FDA rejected approval for quizartinib because of major doubt about the limited survival benefit shown in the QuANTUM-R trial.

### Gilteritinb

The highly selective type I next-generation FLT3-inhibitor gilteritinib was analyzed in a phase I–II trial in seven dose escalation cohorts of relapse/refractory AML and showed favorable safety profile and consistent FLT3 inhibition in most patients receiving a daily dose of 80 mg or higher [38]. Based on the promising CRc rates of 41% of FLT3-ITD-mutated AML patients receiving at least 80 mg/day, gilteritinib in the dose of 120 mg/day was compared to salvage chemotherapy in a randomized controlled phase III clinical trial (ADMIRAL) including 371 FLT3-mutated relapse/refractory AML patients [14]. Gilteritinib monotherapy resulted in a significantly higher composite complete remission rate compared to salvage chemotherapy (54.3% vs 21.8%, HR 32.5, 95% CI 22.3–42.6), higher overall survival (median 9.3 months vs 5.6 months, *p* < 0.001) and event-free survival (median 2.8 months vs 0.7 months, HR 0.79, 95% CI 0.58–1.09). Compared to other inhibitors, gilteritinib had a better safety profile as severe adverse events occurred less frequently in the gilteritinib group compared to the salvage therapy group. Seventy-four patients included in the study underwent gilteritinib regimen for relapse after allo-HCT and, interestingly, relapse in the first 6 months after allo-HCT was associated with a high benefit from gilteritinib therapy compared to salvage chemotherapy (HR for death 0.38, 95% CI 0.20–0.75). Based upon these results, gilteritinib was approved by the FDA for relapsed/refractory AML in 2018. These findings were confirmed in the Japanese subpopulation in an open-label phase I trial [39], and in a recent subgroup analysis of the ADMIRAL trial [40]. Of note, also in the ADMIRAL trial, patients who previously received FLT3-TKI represent only 12.4%, making it difficult to apply to the current population receiving midostaurin as standard treatment.

Based on the high response rates, gilteritinib may be the most promising FLT3-TKI and ongoing trials are testing gilteritinib in combination with venetoclax and hypomethylating agents in relapsed/refractory AML (NCT03404193, NCT03404193). The role of TKI in post-allo-HCT setting is not tested here as the number of patients with post-transplantation relapse is usually very low in these studies: Maiti and colleagues included only 4 transplanted patients in their phase II trial on triple combination therapy with gilteritinib–venetoclax–decitabine, but obtained a promising CRc of 62% in relapse/refractory patients [41].

### Midostaurin

Despite the defined role of midostaurin in first-line therapy of FLT3-ITD-mutated AML [13], this first-generation kinase inhibitor has not been intensively studied as salvage therapy in the context of post-transplant relapse.

## Maintenance therapy

Based on the promising results in relapse/refractory AML, treatment with FLT3-TKI was tested as maintenance therapy in clinical trials to elucidate its potential in preventing the occurrence of relapse after allo-HCT. Phase II–III trial on maintenance treatment after allo-HCT are listed in Table 2.

**Table 2:** Phase II and III trials on maintenance therapy with FLT3 inhibitor in FLT3-ITD-mutated AML

Inhibitor	Phase	Reference	Patients	Tretment	relapse rate	survival
Midostaurin	Phase II (RADIUS)	Marziarz 2020 NCT01883362	60 (18–70 years)	12 months	n.a.	RFS 18 m: 89% vs 76% (*p* = 0.27) OS 2 years: 85% vs 76% (*p* = 0.34)
Sorafenib	Phase II (SORMAIN)	Burchert 2020 DRKS00000591	83 (18–75 years)	24 months	n.a.	RFS 24 m: 53% vs 85% (*p* = 0.002) OS 2 years: 90.5% vs 66.2% (*p* = 0.007)
	Phase III	Xuan 2020 NCT02474290	202 (18–60 years)	6 months	1 year rel.: 7% vs 24% (*p* = 0.001)	RFS 24 m: 78.9% vs 56.6% (*p* < 0.0001) OS 2 years: 82.1% vs 68.0% (*p* = 0.012)
Gilteritinib	Phase III	NCT02997202	356 (> 18 years)	24 months	n.a. (trial ongoing)	n.a. (trial ongoing)
Crenolanib	Phase II	NCT02400255	target 48 (> 18 years)	24 months	n.a. (trial ongoing)	n.a. (trial ongoing)

*OS* overall survival, *RFS* relapse-free survival, *n.a.* not available

### Midostaurin

Induction therapy and maintenance with Midostaurin in FLT3-ITD-mutated AML had shown a very low relapse rate [42, 43], though, clinical data on maintenance after allo-HCT are very few. A recent phase II randomized open-label trial including 60 patients, included patients in CR1 after allo-HCT that were then randomized to receive maintenance with midostaurin or not. The study demonstrated safety and feasibility when midostaurin was given for 12 months after allo-HCT. Grade 3 adverse event occurred in not more than 10% of patients and, apart from gastrointestinal adverse events, they were comparable in both treatment arms. Although the study was not powered to detect the efficacy, there was a non-significant trend toward a benefit in relapse-free survival (18-months RFS 89% vs 76%) and overall survival (2-year OS 85% vs 76%) in the midostaurin arm compared to standard treatment alone [44].

### Sorafenib

Several early phase studies and case reports have reported evidence for the efficacy of sorafenib maintenance therapy after allo-HCT in patients with FLT3-ITD AML [45–47]. The results of a placebo controlled randomized phase II trial (SORMAIN trial) indicated a higher relapse-free survival (24-months RFS 85% vs 53%, *p* = 0.002) and overall survival (2-year OS: 90.5% vs 66.2%, *p* = 0.007) in the sorafenib group compared to the placebo group [48]. These results were in agreement with a multicenter open-label randomized phase III trial [49], and by several real-world retrospective studies [50–52]. A recent retrospective study reported a higher overall survival and leukemia-free survival in patients receiving sorafenib as maintenance therapy compared to one prophylactic infusion of DLI [53].

The efficacy of sorafenib is connected to side effects. A prospective single-arm pilot study [54] and the work of Morin and colleagues presented at the 62nd annual meeting of the American Society of Hematology showed a rate of up to 90% of drug interruption or dose reduction from “standard dose” of 400 mg bid mainly due to gastrointestinal and hematological (thrombocytopenia, neutropenia) toxicities [55]. Praz et al. suggested therefore a standard dose reduction to 200 mg bid, to be increased based on accurate symptom-guided dosing.

Subgroups analyses to identify variables that correlate with better outcome were conducted in both randomized trials. In the SORMAIN trial, patients who reached a MRD negativity before transplantation showed a higher benefit from sorafenib maintenance, compared to MRD-positive patients [48]. Interestingly, in the study from Xuan and colleagues, sorafenib maintenance showed the strongest benefit in patients who received allo-HCT from matched sibling donor and in patients without acute GVHD, suggesting an immunomodulatory role of maintenance therapy. On the other hand, in a study from Shao and colleagues presented at 63rd annual meeting of American Society of Hematology, the concomitant mutation of CEBPA and TET2 neutralized the positive effect of sorafenib in FLT3-ITD-mutated AML [56].

Based on these studies, the Acute Leukemia Working Party of the European Society for Blood and Marrow Transplantation recommends post-transplant maintenance therapy with a FLT3 inhibitor, preferably sorafenib, for patients who undergo allo-HCT for FLT3-ITD-mutated AML [57].

### Quizartinib

Phase I data on quizartinib show acceptable tolerability and feasibility of FLT3-TKI maintenance after allo-HCT and, even though very preliminary, these data show early evidence of low relapse rate with only one relapse in 13 treated patients (8%) [58].

### Gilteritinib and crenolanib

Currently, a prospective phase III randomized, double-blind, multicenter trial (NCT02997202) using gilteritinib versus placebo for patients with FLT3-ITD AML as maintenance after allo-HCT is ongoing [59]. Stratified results per MRD-status on 365 enrolled patients are expected for 2025. Results of the single-arm phase II study (NCT02400255) using crenolanib as maintenance therapy after allo-HCT are not yet published.

### Open issues in maintenance therapy

A comparison between sorafenib and midostaurin was conducted in a multicenter retrospective cohort study by Shimony and colleagues [60]. Here, even though only 41 patients were included and most received pre-transplantation FLT3-TKI too, maintenance therapy with sorafenib was superior to midostaurin in terms of OS and RFS (HR for survival 0.25, CI 0.07–0.89).

The optimal duration of maintenance therapy is also subject of debate. In the SORMAIN trial on sorafenib and in the ongoing clinical trials on gilteritinib and crenolanib, the maintenance therapy was conducted for up to 2 years. Midostaurin was administered in the trial for 12 months. Xuan and colleagues reduced the period of sorafenib treatment to 6 months claiming concerning about risk of developing drug resistance. Currently, the Leukemia Working Party recommends a duration of 2 years for maintenance therapy, to be adapted to tolerability [57].

One last frequently debated issue is the need of treatment of MRD positivity. FLT3 inhibitors offer a valid and relatively safe therapy in the context of MRD positivity after transplantation. Both the phase II [48] and the phase III trial [49] showed a benefit of sorafenib maintenance therapy also in MRD-positive patients after allo-HCT, with a reduction of relapse incidence at 2 years of 44% (33.3% vs 77.3% in sorafenib vs control group, HR 0.25, 95% CI 0.06–094) [49]. Since there is no randomized clinical trial to address the question which FLT3-TKI is most effective as maintenance therapy for FLT3-ITD AML, a definitive response cannot be given. However, using a TKI to reduce the risk of relapse is recommended [57].

## Preclinical evidence for a role in anti-tumor immunotherapy

As previously discussed in this review, sorafenib showed clinical evidence of synergism with allo-immunity against leukemia cells and enhanced graft-versus-leukemia effect. These synergism-motivated studies in mouse models which showed that FLT3 inhibition combined with T cell infusion can lead to complete elimination of leukemia cells. Mechanistically, FLT3 inhibition reduced the expression of the transcription factor ATF4. ATF4 normally blocks interferon regulatory factor 7 (IRF7) activation and this effect was antagonized by the FLT3 inhibition. Taking away the ATF4-mediated blockade, sorafenib allowed IRF7 activation and caused IL-15 transcription in the leukemia cells which caused an increase of CD8+ CD107a+ IFNγ+ T cells, which was connected to the increased elimination of leukemia cells by activated donor T cells [20] (Fig. 1).

Increased IL-15 was detected in the blood of responder patients and caused increased mitochondrial spare respiratory capacity in T cells, which is consistent with previous reports indicating that IL-15 causes mitochondrial reprogramming in T cells [61, 62]. Sorafenib, however, is a multi-tyrosine kinase inhibitor. To answer the question if also a selective FLT3 inhibitor could enhance GvL-effect, Zhang and colleagues studied IL-15 production and T cell activation in a mouse model of FLT3-ITD-positive AML relapse after allo-HCT treated with gilteritinib with or without T lymphocyte infusion [21]. Upon FLT3 inhibition, an increased production of IL-15 and a reduction of exhaustion T cell marker could be identified. Interestingly, a short-term administration of gilteritinib alone was not able to suppress leukemia growth in the absence of T lymphocytes [21].

## Development of resistance

Within few months from initial response, the development of resistance to FLT3 inhibitors can occur and this typically limits the use of FLT3-TKI as monotherapy. The first predicted mechanism of resistance against type II inhibitors is the on-target mutation of TKD at activation loop D835 or at gatekeeper F691 [17]. Accordingly, these and other on-target mutations had been found in patients treated with quizartinib and sorafenib [16, 19, 63, 64]. However, these mutations cannot explain the occurrence of resistance to type I inhibitors and the fact that only a small proportion of patients presents on-target mutations at relapse. Interesting recent next-generation sequencing analysis on AML at relapse after gilteritinib [65], midostaurin [66] and crenolanib [18] revealed the impact of clonal heterogeneity on the development of resistance to selective FLT3 inhibition in AML. One common underlying mechanism is the acquisition of activating mutations on alternative pathways, such as NRAS/KRAS [65], AXL [67], JAK-family [68], PTPN11, KIT, NF1s [66], leading to an alternative way to STAT5 phosphorylation in the same original leukemic clone or alternatively, in a FLT3-ITD independent sub-clone. These findings open the potential of multiple targeted therapies to overcome occurrence of relapse and, together with the broad inhibitory activity against multiple kinases, presumably explain the efficacy of re-challenging with a second FLT3 inhibitor after initial TKI-relapse. Accordingly, gilteritinib recently showed similar responses in patients pre-treated with midostaurin [69]. Activation of alternative pathways, such as KRAS/RAC1/ROS/NLRP3, in leukemia cells [70, 71] or non-hematopoietic cells [72] may also lead to the production of pro-inflammatory IL-1β that promotes leukemia cell proliferation in an autocrine fashion.

## Summary and outlook

Based on the results obtained in clinical trials, FLT3-inhibitor maintenance is the recommended therapy for FLT3-ITD AML after allo-HCT. Gilteritinib has shown remarkable efficacy and acceptable toxicity in FLT3-ITD AML patients outside of the allo-HCT setting. However, since the ADMIRAL trial was conducted before the addition of midostaurin to induction therapy became standard of care, gilteritinib’s role in relapsed/refractory disease has to be re-evaluated carefully. For post-allo-HCT maintenance therapy, the Leukemia Working Party recommends sorafenib based to the large amount of available data and the stimulation of GvL effect. The ongoing trials on gilteritinib as maintenance and as combination therapy will clarify the importance of FLT3 inhibitors in this clinical context. In future, more data are needed to further unveil the potential advantages of these multi-kinase inhibitors, to understand and overcome possible relapse mechanisms and to fine-tune the immunogenic capacity through synergism with allo-HCT and combination with DLI.
